# Bullous Varicella in an Immunocompetent Adult: A Case Report

**DOI:** 10.7759/cureus.83510

**Published:** 2025-05-05

**Authors:** Varsha R Parupati, Arun Vignesh, Anuradha Priyadarshini, Murugan Sundaram, Dr Adikrishnan Swaminathan

**Affiliations:** 1 Dermatology, Venereology, and Leprosy, Sri Ramachandra Institute of Higher Education and Research, Chennai, IND

**Keywords:** chickenpox, staphylococcal epidermolytic toxin, tzanck smear, varicella bullosa, varicella infection

## Abstract

Chickenpox is a common viral infection that usually runs a benign course. Bullous varicella is an atypical presentation of chickenpox, which is usually seen in immunocompromised individuals. We describe a 54-year-old immunocompetent woman who presented to the emergency room with a 10-day history of fever and painful, fluid-filled lesions on her face, trunk, and extremities. Upon examination, the face, trunk, and extremities showed many vesicles, pustules, and bullae over an erythematous base. Multinucleated giant cells (MNGs) with acantholytic cells were visible in the Tzanck smear. A diagnosis of bullous varicella was made based on the appearance of the lesions and the results of the Tzanck smear. The patient was treated with intravenous acyclovir and antibiotics. We report this case due to the rarity of bullous varicella in immunocompetent adults.

## Introduction

Varicella infection, more commonly called chickenpox, is a viral infection commonly seen in children, during late winters and early summer [1]. It is caused by the varicella zoster virus (VZV) [2]. Typically, the illness progresses in a benign and self-limiting manner. It is characterized by the development of fever and a pruritic vesicular rash [3]. Occasionally, atypical presentations such as varicella gangrenosa, bullous varicella, varicella neonatorum, and hemorrhagic varicella may be seen [1]. Bacterial superinfection is one of the complications that can occasionally result in cellulitis, necrotizing fasciitis, toxic shock syndrome, and staphylococcal scalded skin syndrome [2,3].

One unusual manifestation of a VZV infection is bullous varicella. It is usually seen in immunocompromised individuals. It is characterized by the presence of large flaccid hemorrhagic bullae, which rupture and form erosions. Although the exact etiology is unknown, the staphylococcal epidermolytic toxin is believed to be the underlying cause [1]. Although this condition does not substantially alter the overall prognosis of VZV infection, it plays an important role in guiding treatment, necessitating the addition of antibiotics alongside standard antiviral therapy. Early recognition would also help in preventing complications like sepsis.

In any case of VZV infection presenting with atypical manifestations, it is pertinent to rule out an immunocompromised state. We present a case of bullous varicella in a 54-year-old immunocompetent woman, highlighting its rarity in adults, especially those who are immunocompetent.

## Case presentation

**Figure 1:**
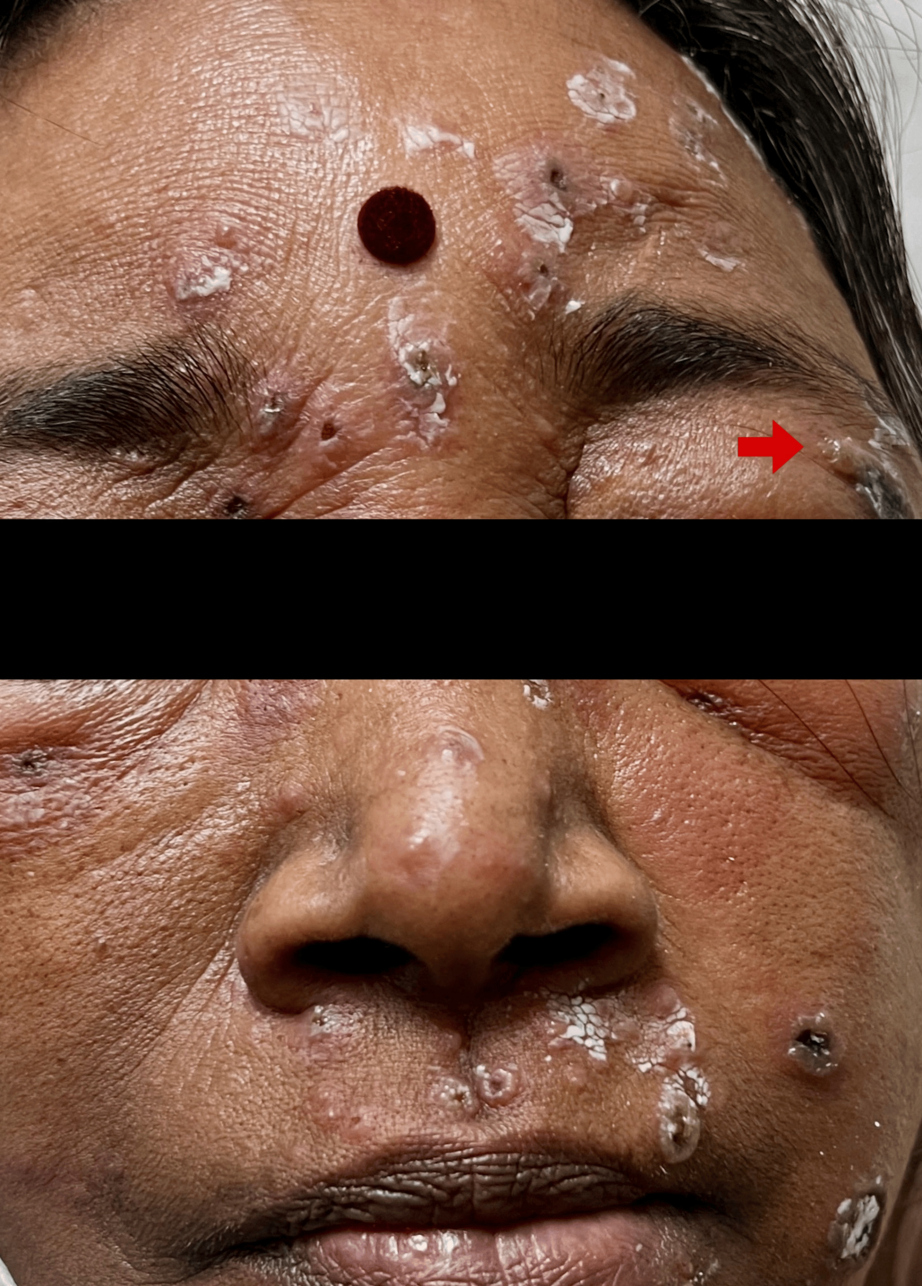
Multiple vesicles, pustules, and crusted plaques noted over the face. Red arrow depicting a single 1.5 cm x 1 cm bulla over the left upper eyelid.

A 54-year-old woman presented with a 10-day history of multiple fluid-filled lesions on the face, trunk, and extremities. The lesions were pruritic and ruptured spontaneously to form painful erosions. The patient reported fever with chills since the onset of the rash, along with periorbital swelling that had been present for the past five days. No history of drug intake, vaccination, or native medication intake was noted before the onset of the rash. The patient denied any history of mucosal involvement, recent travel, or contact with a chickenpox case. Additionally, there was no history of prior chickenpox infection or vaccination. On examination, the patient was stable, and the systemic examination was found to be normal. Multiple clear vesicles and bullae, along with a few pustules, were observed on an erythematous base across the face, trunk, and extremities, some of which exhibited an annular configuration (Figure 1).

**Figure 2:**
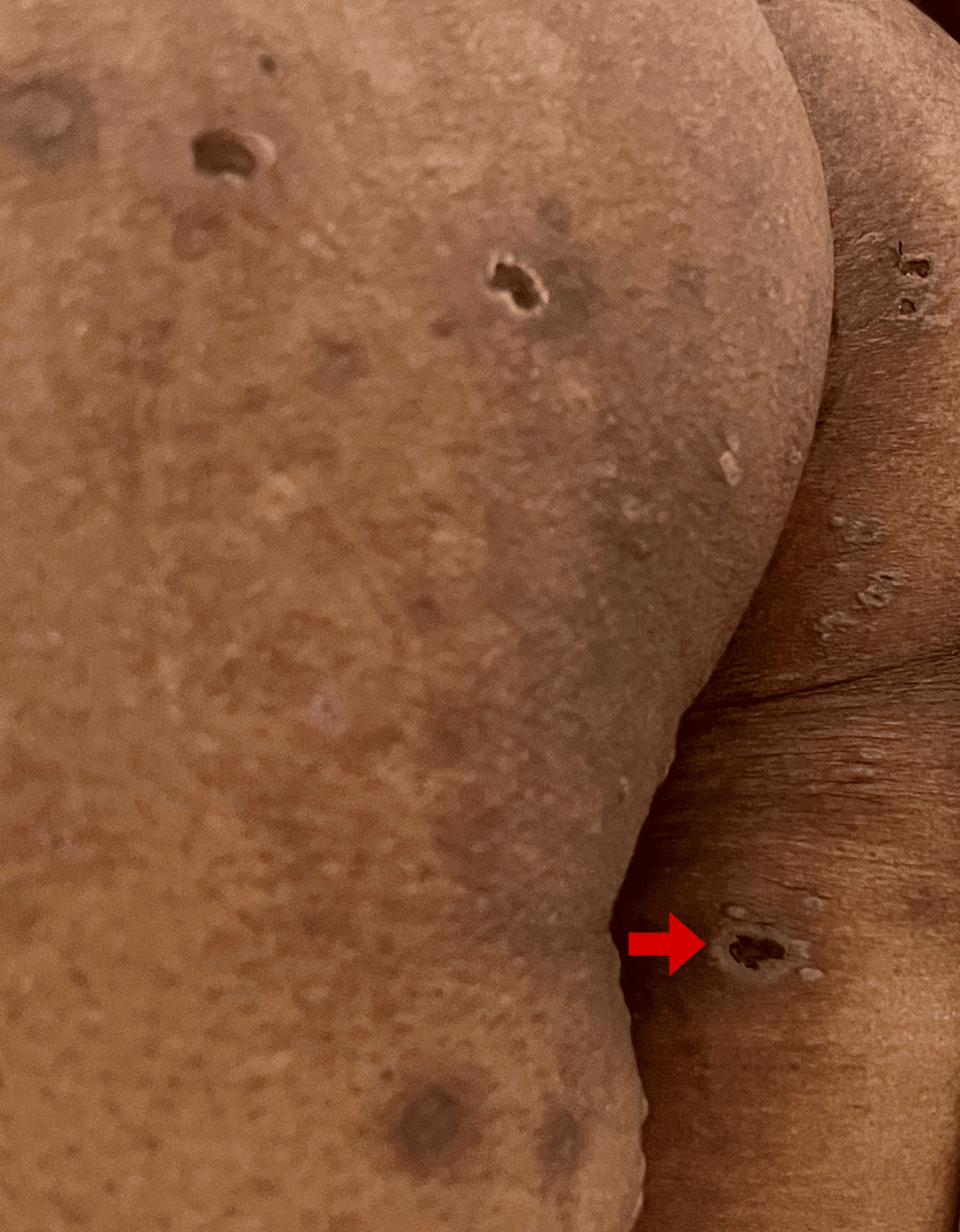
Multiple crusted plaques noted over the gluteal region and lower extremities. Red arrow showing a crusted plaque with surrounding pustules on the posterior aspect of the right thigh.

Multiple discrete erythematous tender papules and crusted plaques were noted over the trunk, gluteal region, and extremities (Figure 2).

**Figure 3:**
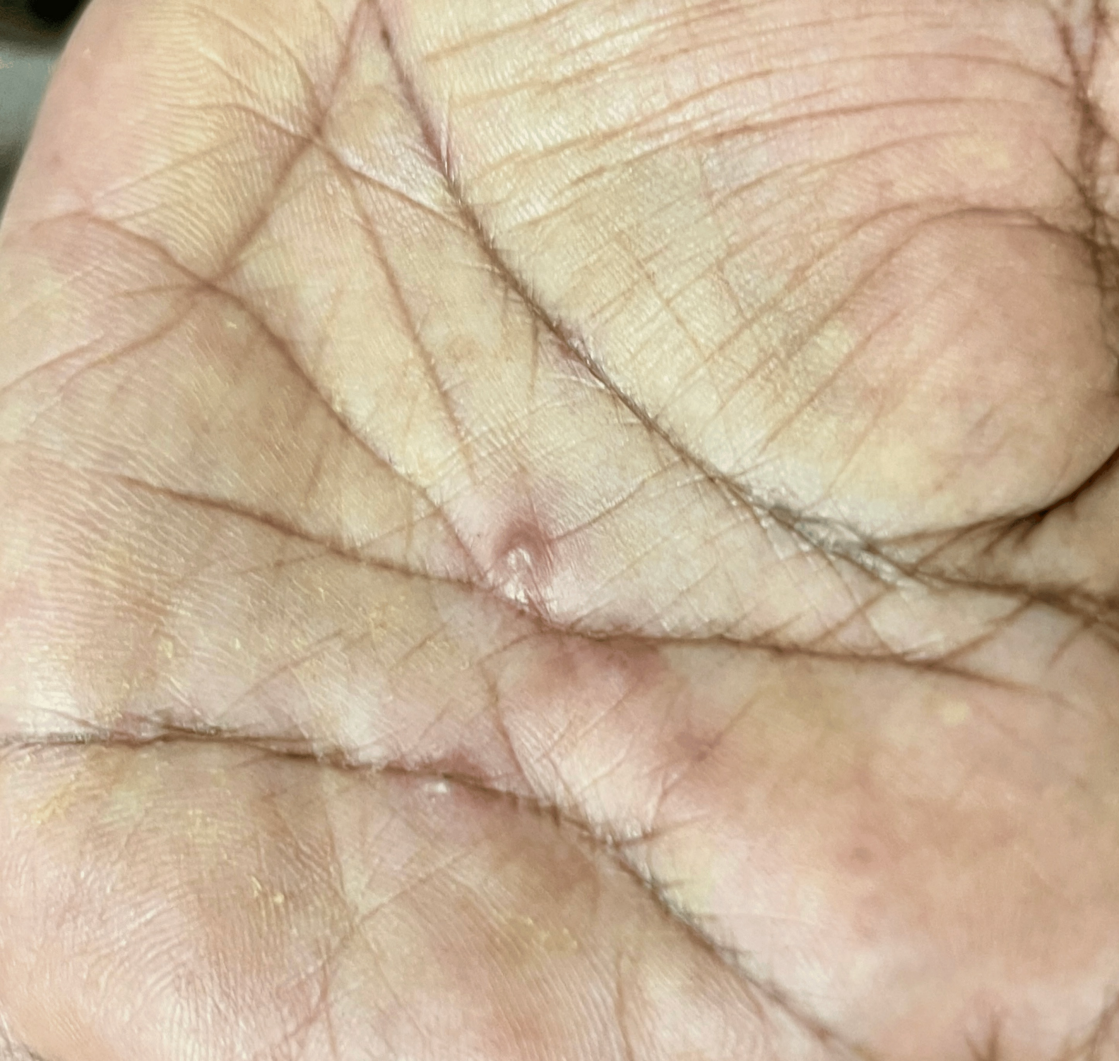
The palm showing a few deep-seated vesicles.

Palmar involvement was seen in the form of deep-seated vesicles (Figure 3).

**Figure 4:**
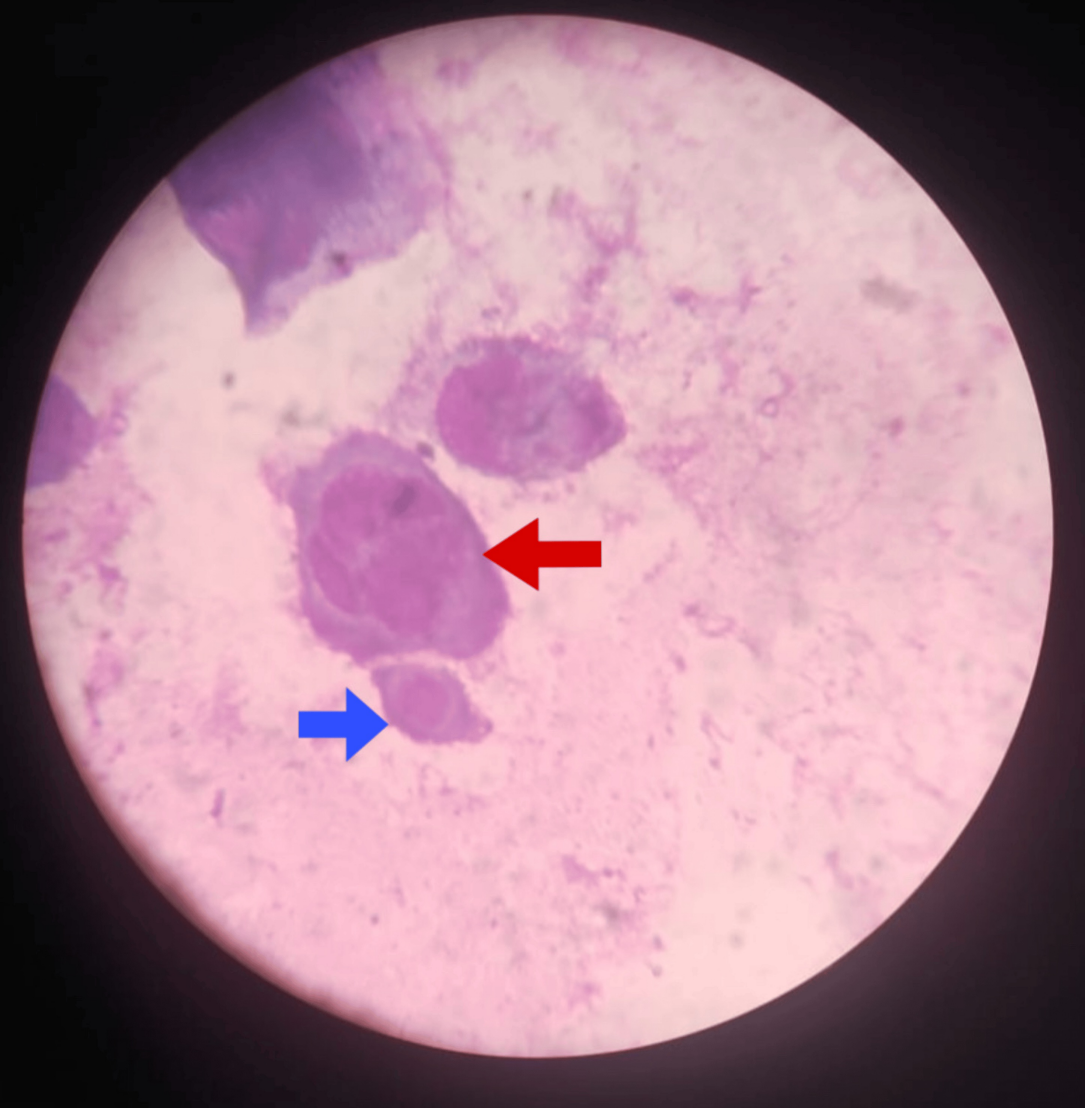
Tzanck smear showing an acantholytic cell (blue arrow) and a multinucleated giant cell (MNG, red arrow).

Bilateral periorbital edema was noted. Examination of the oral cavity revealed a few erosions on the gingival mucosa. Nikolsky's sign was negative. Based on the presenting features, erythema multiforme (EMF) and linear IgA bullous dermatosis (LABD) were considered. Routine investigations revealed an elevated total leukocyte count of 12,510 cells/mm³, with mild neutrophilia and lymphopenia. Blood and swab cultures were negative for any microbial growth. The patient tested negative for HIV, syphilis, hepatitis B, and hepatitis C. No other symptoms, signs, or investigations indicated an immunocompromised state. A Tzanck smear from one of the vesicles revealed multinucleated giant cells and acantholytic cells (Figure 4).

These findings confirmed the diagnosis of bullous varicella. The patient was initiated on intravenous acyclovir 250 mg three times daily, as advised by the nephrologist due to mildly elevated serum creatinine levels, along with intravenous amoxicillin-clavulanic acid 1.2 g twice daily for seven days. Supportive therapy was also provided, resulting in significant clinical improvement. At the 10-day follow-up, the patient demonstrated complete recovery with only minimal post-inflammatory hyperpigmentation.

## Discussion

Varicella infection is a commonly occurring viral infection caused by VZV, which belongs to the human alpha herpesvirus group [3]. It classically presents with a pruritic, polymorphous eruption of macules, papules, and vesicles, typically showing a centripetal spread. Lesions usually appear in successive crops, with the classical lesion being a clear vesicle on an erythematous base, described in the literature as having a *dewdrop on a rose petal* appearance [1].

Although the classical presentation of varicella infection is easily diagnosed, aided by bedside diagnostic tests like the Tzanck smear, which reveals MNGs, atypical presentations are becoming increasingly common. Prompt diagnosis and treatment are important in such cases.

Bullous varicella is one such atypical presentation. The development of flaccid bullae characterizes bullous varicella, due to the production of staphylococcal epidermolytic toxin [4]. These bullae often become hemorrhagic and rupture, leaving raw areas. Sepsis is a significant complication, typically caused by Streptococcus pyogenes and Staphylococcus aureus. Impetigo, lymphadenitis, scarlet fever, cellulitis, erysipelas, bacteremia, septic arthritis, and pneumonia are other commonly encountered complications [4]. Scarring may occur as a result of secondary bacterial infection [2].

Bullous lesions do not affect the disease course, prognosis, or serve as an indicator of disease severity [5].

Other atypical manifestations include varicella gangrenosa, hemorrhagic varicella, Stevens-Johnson syndrome, and EMF [6]. These atypical manifestations have been primarily reported in immunocompromised children and, rarely, in adults. Vaccinated individuals are less likely to present with atypical manifestations [3]. This case highlights the importance of considering bullous varicella even in immunocompetent adults, as treatment with antivirals and anti-staphylococcal medications leads to a good prognosis. Acyclovir is the most commonly used antiviral, administered at a dosage of 10 mg/kg per dose, given intravenously three times daily. Other antiviral options include valacyclovir and famciclovir.

Sinha et al. [1] reported a case of bullous varicella in a 21-year-old male. de Salles-Gomes [7] reported a case of bullous varicella in a White male, with lesions occurring over areas irradiated by sunlight. Other cases have predominantly been diagnosed in infants and children, as reported by Mansouri et al. [2] in a six-month-old immunocompetent boy, by Gor et al. [3] in a seven-year-old girl with type 1 diabetes mellitus, celiac disease, and a history of COVID-19, and by Kurban et al. [8] in a healthy nine-year-old boy. Case reports of bullous varicella in immunocompetent adults are scarce.

Other differential diagnoses to be considered in such cases are EMF, bullous insect bite reactions, and immunobullous disorders [8]. In our case, because of the presence of a few lesions resembling a *string of pearls* pattern, we also considered the diagnosis of LABD. Therefore, it is important to differentiate these conditions with a high degree of clinical suspicion, using bedside tests such as the Tzanck smear and Gram staining, and, if necessary, a biopsy with direct immunofluorescence. Early diagnosis aided by simple bedside tests can help avoid unnecessary investigations like VZV polymerase chain reaction, skin biopsy, and direct immunofluorescence.

## Conclusions

Bullous varicella is a rare entity that is often misdiagnosed in the early stages, leading to delays in appropriate management and potentially increasing the risk of complications, such as scarring. Early recognition is essential to distinguish it from other bullous dermatoses and initiate timely antiviral and antibacterial therapy. Most reported cases occur in the pediatric population, especially among those who are immunocompromised. We present this case due to the rare occurrence of bullous varicella in adults, particularly in immunocompetent individuals, where such presentations are exceedingly uncommon. This case report emphasizes the importance of suspecting unusual presentations of varicella, such as bullous varicella, even in otherwise healthy individuals, and highlights the critical need for prompt treatment, including both antibiotics and antivirals. Early recognition, aided by bedside tests such as the Tzanck smear, facilitates prompt initiation of treatment and helps prevent unnecessary diagnostic investigations.

## References

[1] Sinha P, Bhattacharjee S, Chatterjee M (2017). Varicella bullosa in an adult. Med J Armed Forces India.

[2] Mansouri S, Mai S, Hassam B, Benzekri L (2019). Bullous varicella in an immunocompetent infant. BMJ Case Rep.

[3] Gor NM, Mevawala HB, Tanna SS, Ribadiya FN, Potapchik A (2024). Varicella bullosa: a rare form of chickenpox in an immunocompromised child—a case study. Int J Res Med Sci.

[4] Sathyanarayana BD (2025). Varicella bullosa. Indian J Dermatol Venereol Leprol.

[5] Beniwal R, Gupta LK, Khare AK, Mittal A, Mehta S, Balai M (2018). Varicella masquerading as pemphigus vulgaris. Indian J Paediatr Dermatol.

[6] Gangadharan G, Criton S (2024). An unusual presentation of varicella. J Turk Acad Dermatol.

[7] de Salles-Gomes LF (1981). Anomalous clinical pictures of varicella. Int J Dermatol.

[8] Kurban M, Saleh Z, El Shareef M, Kibbi AG, Ghosn S (2008). Bullous chickenpox: an unusual clinical variant of varicella. Int J Dermatol.

